# Exploring the Relationship Between Anxiety, Depression, and Sleep Disturbance Among HIV Patients in China From a Network Perspective

**DOI:** 10.3389/fpsyt.2021.764246

**Published:** 2021-10-22

**Authors:** Ni Wang, Muyu Wang, Xin Xin, Tong Zhang, Hao Wu, Xiaojie Huang, Honglei Liu

**Affiliations:** ^1^School of Biomedical Engineering, Capital Medical University, Beijing, China; ^2^Beijing Key Laboratory of Fundamental Research on Biomechanics in Clinical Application, Capital Medical University, Beijing, China; ^3^Center for Infectious Diseases, Beijing You'an Hospital, Capital Medical University, Beijing, China

**Keywords:** network analysis, people living with HIV (PLWH), anxiety, depression, sleep disturbance, hospital anxiety and depression (HAD) scale, pittsburgh sleep quality index (PSQI) questionnaire

## Abstract

**Background:** Mental disorder of people living with HIV (PLWH) has become a common and increasing worldwide public health concern. We aimed to explore the relationship between anxiety, depression, and sleep disturbance for PLWH from a network perspective.

**Methods:** The network model featured 28 symptoms on the Hospital Anxiety and Depression scale questionnaire and Pittsburgh Sleep Quality Index questionnaire in a sample of 4,091 HIV-infected persons. Node predictability and strength were computed to assess the importance of items. We estimated and compared 20 different networks based on subpopulations such as males and females to analyze similarities and differences in network structure, connections, and symptoms.

**Results:** Several consistent patterns and interesting differences emerged across subgroups. Pertaining to the connections, some symptoms such as S12–S13 (“*sleepy”*—“*without enthusiasm”*) shown a strong positive relationship, indicating that feeling sleepy was a good predictor of lacking enthusiasm, and vice versa. While other symptoms, such as A3–D3 (“*worried”*—“*cheerful”*), were negatively related in all networks, revealing that nodes A3 and D3 were bridge symptoms between anxiety and depression. Across all subgroups, the most central symptom was A7 “*panic”* and S2 “*awake”*, which had the greatest potential to affect an individual's mental state. While S3 “*bathroom”* and S5 “*cough or snore”* shown consistent lower node importance, which would be of limited therapeutic use.

**Conclusions:** Mental conditions of PLWH varied considerably among subgroups, inspiring psychiatrists and clinicians that personalized invention to a particular subgroup was essential and might be more effective during treatment than adopting the same therapeutic schedule.

## Introduction

According to the National Health Commission of the People's Republic of China, there were approximately 958 thousand people living with HIV (PLWH) as of October 2019 (1). By the end of 2020, nearly 19 thousand people died of AIDS in China, which was the disease with the highest number of reported death (2). Living with HIV implies facing discrimination, perceiving stigma, lacking support, and having sexual problems. Thus this population usually have a high prevalence of mental health problems such as anxiety, depression, sleep disturbance, and autism and so on (3). Previous studies have reported that over half of all PLWH suffer from mental health disorders (3, 4). The mental disorders are usually related to a wide range of negative outcomes such as decreased quality of life, poor psychosocial functioning, and high drug use (3, 5). This problem thus has become a common and increasing worldwide public health concern.

However, to date, this issue has been largely ignored and insufficiently addressed in global policy guidelines (6). In China, the treatment-seeking rate is <6% for disorders such as anxiety and substance use disorders among general population, not to mention PLWH (7, 8). In this line, there is a substantial need for mental health services as well as the development of successful targeted programs for psychological support and care among PLWH. Many researchers have tried to understand the mental disorder burden and seek treatment for PLWH. For instance, Huang et al., (9) conducted a cross-sectional study in China, attempting to identify risk factors of anxiety and depression by using the logistic regression analysis for PLWH. Rihs et al., (10) demonstrated that patients prescribed the efavirenz had experienced severe to extremely severe anxiety, depression, and stress. Moskowitz et al., (11) conducted a randomized controlled trial to identify whether a positive affect skills intervention could improve emotion and psychological health in people newly diagnosed with HIV. Chen et al., (12) used a qualitative and biomedical approach to explore how sleep and energy levels were affected in women diagnosed with HIV in China. Lee et al., (13) conducted a cross-sectional study to characterize specific types of sleep problems experienced by adults with HIV.

In recent years, the network approach to psychopathology has been proposed as a novel way of analyzing mental disorders (14). It has been successfully applied to many disorders such as the posttraumatic stress disorder (15), depression (16), substance abuse and dependence (17), and autism (18) and so on. The previous network studies usually examined symptom relationships and centrality within a single disorder. Few of them attempted to analyze co-morbidity, which was extremely common in the realm of psychopathology (19). Prior studies have demonstrated that patients with multiple disorders at the same time usually have poorer prognosis, worse treatment outcomes, and higher suicide rates (20). Under this situation, we attempted to explore the relationships between three common mental disorders (i.e., anxiety, depression, and sleep disturbance) for PLWH by using the network model. The network model can portray a structure describing how all components interact, which is an ability not available to traditional common cause models. Thus we conducted a network analysis herein to represent several disorders as a web of mutually influencing symptoms attempting to provide promising leads toward clinical prevention and intervention improvement. We believed the current study could provide novel insights into the complex relationships between anxiety, depression, and sleep disturbance. To the best of our knowledge, this was the first network analysis for the three mental disorders of PLWH.

## Methods

### Participants

Data for the analyses carried out in the present study came from 4,101 HIV-infected persons who participated in a cross-sectional study among 20 HIV treatment clinics across China. All enrolled participants completed the Hospital Anxiety and Depression (HAD) scale questionnaire (21) and the Pittsburgh Sleep Quality Index (PSQI) questionnaire (22). The two questionnaires were translated into Chinese for participants self-rating. The HAD scale questionnaire consists of seven items on depression (HAD-D) and anxiety (HAD-A), respectively. They both use a four-point response scale such as ‘*0*=*Not at all; 1* = *Sometimes; 2* = *Very often; 3* = *Nearly all the time'*. The PSQI questionnaire is composed of 18 items measuring the quality and patterns of sleep. Among them, 14 items are scored on a 0–3 scale, wherein three reflects the negative extreme of sleep disorders. Each item in the questionnaire is a symptom from a clinical standpoint and is represented as a node in the network model.

Demographics and behavioral data were also collected through the self-administered survey. Clinical data were extracted from medical records, including current CD4+ cell counts and current HIV RNA levels. Therefore, a total of 11 factors, namely age (18~35 years and >35 years), gender (male and female), education (< high school, high school, and >high school), marital status (married and single), employment (student, blue collar, and white collar), transmission route (homosexual and heterosexual), HIV status disclosed to friends (yes and no), HIV status disclosed to family (yes and no), support from society/family (with and without), current CD4+ cell counts (≤ 350 cells/mm^3^ and >350 cells/mm^3^), and HIV RNA levels (<50 copies/mL and ≥50 copies/mL) were included in the present study as baseline characteristics for downstream analyses. This study was approved by the Beijing You'an Hospital institutional review board. All participants provided written informed consent to complete a survey and extract their clinical data from medical records.

### Statistical Analyses

The rating scores of all items in HAD-A/D were summed and the total score ≥8 on the anxiety and depression items indicated positive signs for anxiety and depression (21). The participants with a score >5 on PSQI were considered to be at high risk of sleep disturbance (22). We used the Pearson χ^2^-tests to compare proportions among the abovementioned 11 factors and the three mental disorders (i.e., anxiety, depression, and sleep disturbance). All *P*-values were two-sided, and *P* < 0.05 were considered significant. These analyses were conducted using SPSS 25.0.

### Network Analyses

According to the Pearson χ^2^-tests, we identified the factors showing significant differences in any mental disorders. This univariate statistical inference was intuitive and easy-to-understand without considering pairwise relations of all symptoms in the questionnaire. Thus, we further conducted the network analysis for a comprehensive consideration and thorough comparison in the symptom-level of the HAD and PSQI questionnaires. The software R (version 3.6.1, open source, available at https://www.r-project.org/) was used to carry out the network analysis. Packages and functions used in this study included estimate Network (*bootnet* package) (23) for network construction, glasso (*qgraph* package) (24) for network estimation and visualization, and mgm (*mgm* package) (25) for node predictability.

Due to the requirements of network analysis, 14 items with an ordinal scale in the PSQI questionnaire and all 14 items in the HAD questionnaire were used to build networks in this study. Table 1 provides a detailed description of these items. Note that items A4, D1, D2, D3, D6, and D7, were reverse scored to ensure data quality. For better understanding, we rearranged these items' scores.

**Table 1:** Study items in the hospital anxiety and depression scale questionnaire and the pittsburgh sleep quality index questionnaire.

**Node**	**Item/Symptom**	**Description**
A1	I feel tense or “wound up”	Tense
A2	I get a sort of frightened feeling as if something awful is about to happen	Frightened
A3	Worrying thoughts go through my mind	Worried
A4	I can sit at ease and feel relaxed	Relaxed
A5	I get a sort of frightened feeling like “butterflies” in the stomach	Extreme fear
A6	I feel restless as I have to be on the move	Restless
A7	I get sudden feelings of panic	Panic
D1	I still enjoy the things I used to enjoy	Enjoy things
D2	I can laugh and see the funny side of things	Laugh
D3	I feel cheerful	Cheerful
D4	I feel as if I am slowed down	Dull
D5	I have lost interest in my appearance	No dressing up
D6	I look forward with enjoyment to things	Expect everything
D7	I can enjoy a good book or radio or TV program	Enjoy something
S1	Cannot get to sleep within 30 min	Insomnia
S2	Wake up in the middle of the night or early morning	Awake
S3	Have to get up to use the bathroom	Bathroom
S4	Cannot breathe comfortably	Comfortless breath
S5	Cough or snore loudly	Cough or snore
S6	Feel too cold	Cold
S7	Feel too hot	Hot
S8	Have bad dreams	Dreams
S9	Have pain	Pain
S10	Other reason(s)	Other
S11	During the past month, how often have you taken medicine (prescribed or “over the counter”) to help you sleep?	Medicine
S12	During the past month, how often have you had trouble staying awake while driving, eating meals, or engaging in social activity?	Sleepy
S13	During the past month, how much of a problem has it been for you to keep up enthusiasm to get things done?	Without enthusiasm
S14	During the past month, how would you rate your sleep quality overall?	Self-rate sleep quality

#### Network Estimation

We first estimated one network for each subgroup based on each group's total sample of participants. We then calculated a regularized partial correlation network (26). The Graphical lasso (least absolute shrinkage and selection operator) (24) was used herein to regularize the parameters and avoid the estimation of spurious connections by setting very small edges to zero. Then we visualized the network, wherein an edge's weight represented a regularized partial correlation between two nodes controlling all other nodes in the network. Edges for positive correlations were printed in dark green, and those for negative ones in red. The stronger a connection, the thicker and more saturated an edge was. Finally, we set the maximum edge value across all networks to 0.40 and a minimum value of 0.05 in all networks to enhance the interpretability of the graphs.

#### Network Inference

We further calculated the relative and the absolute importance measure for each node to seek potential risk factors or treatment inventions for mental disorders of the HIV-infected persons. The centrality of nodes, which is used to identify central nodes, is a relative measurement for node importance and a key in network analysis (27–29). There are three common centrality measures: node strength, closeness, and betweenness (30). Node strength is a basic indicator and is often used as a first step when studying networks. It sums all edges of a given symptom with all other symptoms, estimating how strongly a node is directly connected within a network. A node with high strength is likely to activate many other nodes and maybe a good target for intervention from a clinical standpoint (31, 32). Therefore, we used node strength as the main indicator to comment on centrality throughout the article.

We also calculated the node predictability for each node in the networks. Node predictability, an absolute measure of the interconnectedness of a node, is the percentage of the variance of a given node explained by its surrounding nodes (25). It describes the degree to which a given node can be predicted or determined by all other nodes in the network and may help to inform intervention strategies (33). Node predictability is represented in the network as a blue pie chart surrounding the node. Greater node predictability, larger surrounded pie chart.

Generally, we first built network models for all subgroups split according to stratification factors. Then we compared a pair of networks' complexity, structure, and edge weights. Simultaneously, we compared the strength and predictability of each node between the paired networks and analyzed the role it played in different subgroups.

## Results

### Baseline Characteristics

In this study, after excluding six youngsters and four duplicates, we identified a total of 4,091 HIV-infected adults eligible for data analysis. Overall, 78.0% of the participants were male. The enrolled subjects were 18–81 years old (mean ± standard deviation, 38 ± 11.7 years). Approximately 40.0% of participants were infected through anal sex, and 57.1% had a current CD4+ T cell count over 350 cells/mm^3^, and 40.8% were virally suppressed. Over 60% had disclosed their HIV status to their family members, and close to 70% had support from their family members or society. In contrast, just over one-quarter had disclosed their status to a friend (Table 2).

**Table 2:** Characteristics of 4,091 HIV-infected persons (*n*[%]).

**Characteristics**		**HAD-A^#^**			HAD-D^*^			**PSQI^$^**		
		**No anxiety (n=2967)**	**Anxiety (*n* = 1,124)**	***P*-value**	**No Depression (*n* = 2,744)**	**Depression (*n* = 1,347)**	***P*-value**	**No sleep disturbance (*n* = 2,195)**	**Sleep disturbance (*n* = 1,667)**	***P*-value**
Age				0.914			<0.001			0.108
18~35, years	2,024 (49.5)	1,466 (72.4)	558 (27.6)		1,441 (71.2)	583 (28.8)		1,069 (55.6)	792 (41.9)	
>35, years	2,017 (49.3)	1,464 (72.6)	553 (27.4)		1,273 (63.1)	744 (36.9)		1,100 (58.1)	855 (44.4)	
Gender				0.103			0.001			0.643
Male	3,193 (78.0)	2,337 (73.2)	856 (26.8)		2,187 (68.5)	1,006 (31.5)		1,710 (56.7)	1,306 (43.3)	
Female	830 (20.3)	584 (70.4)	246 (29.6)		518 (62.4)	312 (37.6)		450 (57.6)	331 (42.4)	
Education				0.397			<0.001			0.003
< High school	1,527 (37.3)	1,109 (72.6)	418 (27.4)		918 (60.1)	609 (39.9)		870 (60.4)	571 (39.6)	
High school	984 (24.1)	697 (70.8)	287 (29.2)		651 (66.2)	333 (33.8)		518 (55.6)	414 (44.4)	
>High school	1,520 (37.2)	1,114 (73.3)	406 (26.7)		1,135 (74.7)	385 (25.3)		779 (54.2)	657 (45.8)	
Transmission route				0.284			<0.001			<0.001
Heterosexual	1,596 (39.0)	1,186 (74.3)	410 (25.7)		1,043 (65.4)	553 (34.6)		937 (62.5)	563 (37.5)	
Homosexual	1,534 (37.5)	1,114 (72.6)	420 (27.4)		1,096 (71.4)	438 (28.6)		767 (52.9)	684 (47.1)	
Marital status				0.900			<0.001			<0.001
Married	1,695 (41.4)	1,242 (73.3)	453 (26.7)		1,098 (64.8)	597 (35.2)		990 (62.3)	599 (37.7)	
Single	1,709 (41.8)	1,249 (73.1)	460 (26.9)		1,217 (71.2)	492 (28.8)		855 (52.4)	777 (47.6)	
Employment				<0.001			<0.001			0.017
Student	828 (20.2)	552 (66.7)	276 (33.3)		475 (57.4)	353 (42.6)		420 (53.4)	367 (46.6)	
Blue collar	1,558 (38.1)	1,128 (72.4)	430 (27.6)		1,025 (65.8)	533 (34.2)		872 (59.5)	594 (40.5)	
White collar	1,155 (28.2)	867 (75.1)	288 (24.9)		856 (74.1)	299 (25.9)		615 (56.3)	477 (43.7)	
HIV status disclosed to friends				0.132			0.001			0.001
Yes	1,089 (26.6)	809 (74.3)	280 (25.7)		775 (71.2)	314 (28.8)		543 (52.3)	495 (47.7)	
No	2,987 (73.0)	2,148 (71.9)	839 (28.1)		1,959 (65.6)	1,028 (34.4)		1,643 (58.5)	1,167 (41.5)	
HIV status disclosed to family				0.038			0.021			0.029
Yes	2,457 (60.1)	1,812 (73.7)	645 (26.3)		1,615 (65.7)	842 (34.3)		1,350 (58.2)	970 (41.8)	
No	1,623 (39.7)	1,149 (70.8)	474 (29.2)		1,123 (69.2)	500 (30.8)		837 (54.6)	695 (45.4)	
Support from society/family				<0.001			<0.001			<0.001
With	2,797 (68.4)	2,113 (75.5)	684 (24.5)		1,987 (70.9)	813 (29.1)		1,555 (58.8)	1,089 (41.2)	
Without	1,234 (30.2)	816 (66.1)	418 (33.9)		724 (58.7)	510 (41.3)		611 (52.5)	553 (47.5)	
Current CD4+ cell count				0.362			0.321			0.242
≤ 350, cells/mm^3^	1,721 (42.1)	1,235 (71.8)	486 (28.2)		1,139 (66.2)	582 (33.8)		898 (55.7)	713 (44.3)	
>350, cells/mm^3^	2,338 (57.1)	1,708 (73.1)	630 (26.9)		1,582 (67.7)	756 (32.3)		1,729 (57.6)	940 (42.4)	
Current viral load				0.428			0.164			0.900
<50, copies/mL	1,668 (40.8)	1,203 (72.1)	465 (27.9)		1,089 (65.3)	579 (34.7)		885 (56.5)	682 (43.5)	
≥50, copies/mL	1,777 (43.4)	1,303 (73.3)	474 (26.7)		1,200 (67.5)	577 (32.5)		952 (56.5)	733 (43.5)	

# *The total score ≥8 on the HAD-A indicated anxiety*.

* *The total score ≥8 on the HAD-D indicated depression*.

$ *The total score >5 on the PSQI indicated sleep disturbance. Totally 229 participants with missing values on the PSQI were excluded when analyzing sleep disturbance.*

*HAD, Hospital Anxiety and Depression scale questionnaire; PSQI, Pittsburgh Sleep Quality Index questionnaire*.

As shown in Table 2, the sleep disturbance rate of participants whose transmission route was homosexual was about 10% higher than that of participants infected with heterosexuality (*P* < 0.001). On the contrary, the depression rate of participants infected with homosexuality was about 6% lower than that of participants whose transmission route was heterosexual (*P* < 0.001). The HIV-infected persons without spouses also shown a 10% higher sleep disturbance rate than those married (*P* < 0.001). Student infectors with the anxiety and depression rate of 33.3 and 42.6% far exceeded blue collars and white collars (both *P* < 0.001). Furthermore, persons feeling supported by society and family had significantly lower rates of anxiety, depression, and sleep disturbance than those without support (all *P* < 0.001).

There were significant variations in the proportion of depression among all 11 factors except for current CD4+ cell counts and current HIV RNA levels. Actually, no significant variations in the two clinical factors were found among the three mental disorders. Therefore, we excluded the current CD4+ cell counts and HIV RNA levels, leaving nine stratification factors that showed significant variations in the proportion of any mental disorders for further network analyses.

### Item Networks

**Figure 1:**
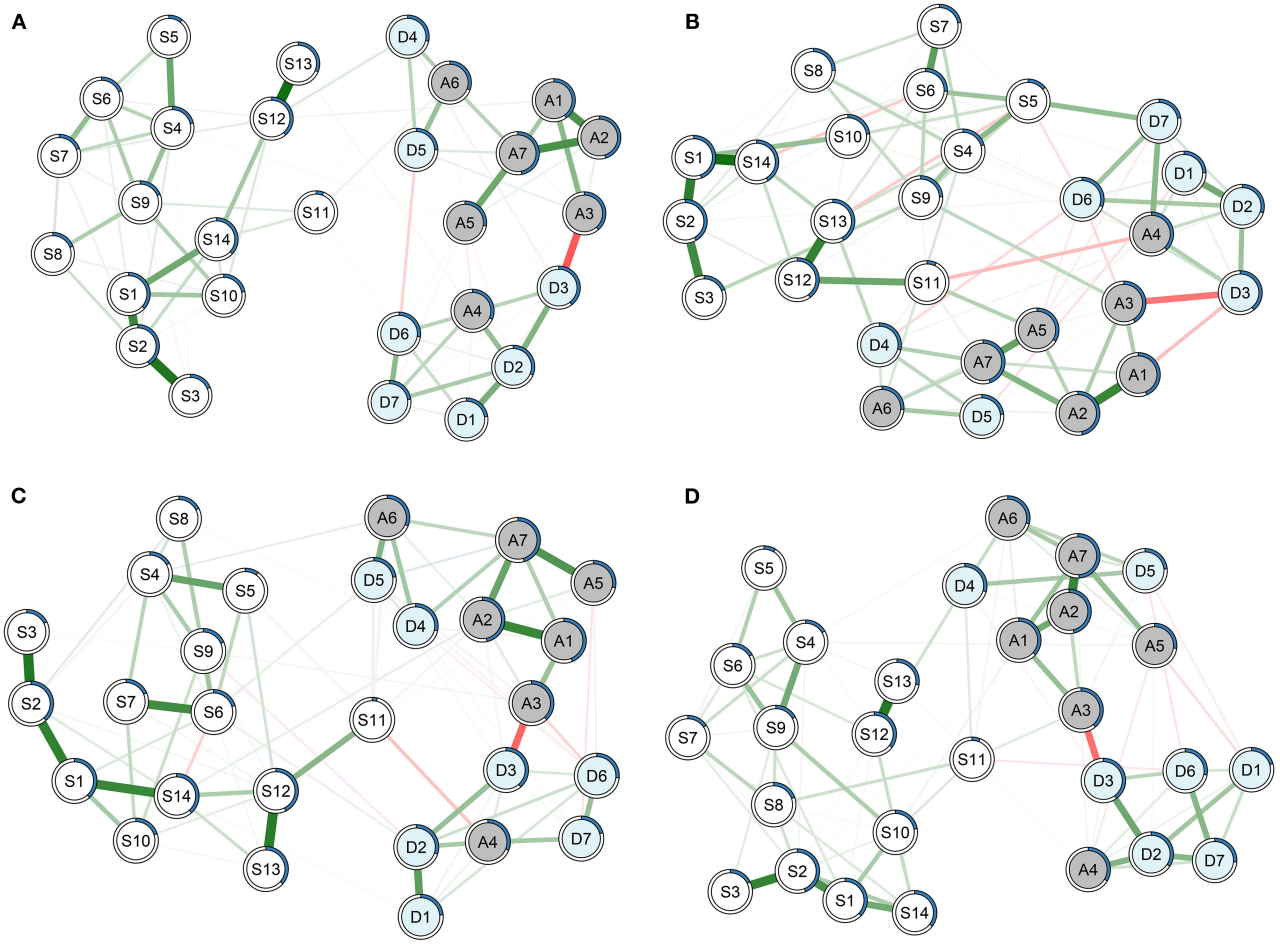
Networks featuring 28 symptoms on the Hospital Anxiety and Depression scale questionnaire and the Pittsburgh Sleep Quality Index questionnaire for **(A)** males, **(B)** females, **(C)** participants infected with heterosexuality, and **(D)** participants infected with homosexuality. Green lines represent positive associations, and red lines negative ones. The thickness and brightness of an edge indicate the association strength. Node predictability is represented as a blue pie chart surrounding the node in the network.

In this section, we built 20 networks for nine stratification factors. Each network featured 28 mental disorder symptoms related to anxiety, depression, and sleep disturbance. Using a network model, we investigated the differences and similarities in mental disorder symptoms between individuals split by each factor. Figure 1 shows two pairs of networks for males and females, and participants infected with heterosexuality and homosexuality. Others can be found in the Supplementary Figure 1.

#### Overview of Networks

The average value of all edges' absolute weights was 0.07 (range, 0–0.40). Going through the 20 networks, several interesting symptom connections were found. For instance, there was a very strong positive connection between nodes S12 ‘*sleepy*’ and S13 ‘*without enthusiasm*’ in each network with an average weight of 0.36 ± 0.026. This result implied that feeling sleepy was a good predictor of lacking enthusiasm, and vice versa. In addition, associations between S1–S2 (‘*insomnia*’—‘*awake*’) and S2–S3 (‘*awake'*—‘*bathroom*’) were presented across 20 networks with the average weights of 0.31 ± 0.031 and 0.33 ± 0.023, separately. Anxiety symptoms A1 ‘*tense*’, A2 ‘*frightened*’, A5 ‘*extreme fear*’, and A7 ‘*panic*’ also showed strong positive connections with the average weights of 0.29 ± 0.028 for A1–A2, 0.27 ± 0.037 for A2–A7, and 0.25 ± 0.023 for A5–A7, respectively, implying a consistent pattern across subgroups.

In contrast, nodes A3 ‘*worried*’ and D3 ‘*cheerful*’ were negatively related with an average weight of −0.25 ± 0.021. This finding mean that nodes A3 and D3 were bridge symptoms between anxiety and depression. Additionally, sleep symptoms were almost completely independent with anxiety and depression symptoms in networks for males, blue collars, participants with support from society/family, and participants who had disclosed their HIV status to family members.

#### Subgroup Analysis

We then estimated and compared networks for each stratification factor to explore the unique patterns of symptom interactions within each subgroup. Apparently, the network built for females with 86 edges was more complex than that for males with 76 edges (Figure 1). Female sleep quality was tightly related to their anxiety and depression symptoms. In addition, there was no edge between nodes S11 ‘*medicine*’ and S12 ‘*sleepy*’ for males and participants with homosexual transmission, indicating that the two nodes were independent controlling for other nodes in the networks. However, the two nodes were strongly connected in networks for females and participants with heterosexual transmission with weights of 0.25 and 0.21, separately. Such difference in association was also found for nodes S5 ‘*cough or snore*’ and D7 ‘*enjoy something*’ between networks for males (weight, 0) and females (0.20).

Additionally, some connections were merely observed in specific subpopulations. For example, nodes A4 ‘*relaxed*’ and S11 ‘*medicine*’ were independent after controlling other nodes in all networks except for those built on females (weight, −0.14) and participants with homosexual transmission (−0.13). Besides, the connection between nodes D2 ‘*laugh*’ and S11 ‘*medicine*’ was merely found in the network built on students with the edge weight of −0.14. Nodes A5 ‘*extreme fear*’ and D6 ‘*expect everything*’ were solely negatively related in the networks built on blue collars (weight, −0.12) and participants that disclosed HIV status to friends (−0.15). Nodes S11 ‘*medicine*’ and S14 ‘*self-rate sleep quality*’ were independent except for the network built on white collars with the weight of 0.19.

### Network Inference

#### Node Centrality

**Figure 2:**
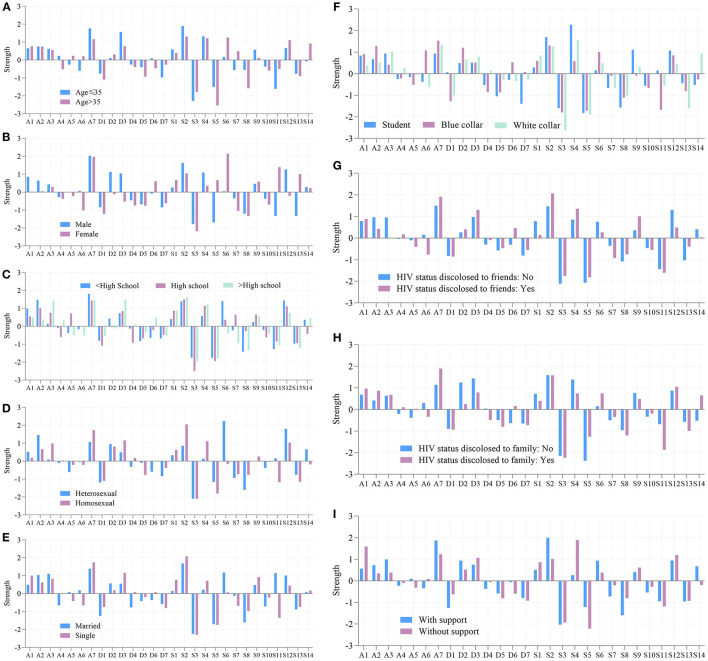
Node centrality of each node in all subgroup networks. **(A)** Age. **(B)** Sex. **(C)** Education. **(D)** Transmission route. **(E)** Marital status. **(F)** Employment. **(G)** HIV status disclosed to friends. **(H)** HIV status disclosed to family. **(I)** Support from society/family. Strength are plotted using standardized z-scores in order to facilitate interpretation.

The standardized estimates of node strength were presented in Figure 2. It can be seen that nodes differ quite substantially in their centrality estimates. Nodes A7 ‘*panic*’ and S2 ‘*awake*’ had consistent high strength among all networks. Meanwhile, nodes S3 ‘*bathroom*’ and S5 ‘*cough or snore*’ showed the lowest strength. Other symptoms, such as S6 ‘*cold*’ varied substantially across networks built on patients with different ages, gender, educations, transmission routes, and marital status in terms of strength.

#### Node Predictability

Some nodes shown consistently higher or lower predictability in all networks, whereas others varied considerably. Node A7 ‘*panic*’ yielded the highest average node predictability of 47% (range, 43–49%), meaning it shared 47% of variance with surrounding items. It was followed by nodes A2 ‘*frightened*’ and S2 ‘*awake*’, with the average predictability of 46% (42–50%) and 41% (38–48%), respectively. It is plausible that an intervention on the surrounding symptoms of panic, frightened, and awake may have a considerable impact on these mental problems. On the contrary, nodes S11 ‘*medicine*’, S5 ‘*cough or snore*’, and S3 ‘*bathroom*’ shown consistent lower node predictability.

Besides, some nodes had quite a different node predictability when estimated in different item networks. Node predictability of S4 ‘*comfortless breath*’ in the network for participants without support from society/family was twice as much on those with support (31 vs. 16%). Also, variations were found in networks built on participants with different employments. For instance, node predictability of A1 ‘*tense*’ varied from 37–48% among networks built on students, blue collars, and white collars, as well as D1 ‘*enjoy things*’ (19–31%), S1 ‘*insomnia*’ (35–45%), S2 ‘*awake*’ (38–48%), S4 ‘*comfortless breath*’ (18–29%), and S13 ‘*lack of enthusiasm*’ (28–40%). Supplementary Tables 1, 2 list all predictability of nodes representing items in HAD questionnaire and PSQI questionnaire, respectively.

## Discussion

In this study, we conducted a network analysis to provide an analytic framework, aiming to examine the association among symptoms of anxiety, depression, and sleep disturbance in PLWH. This was, to our knowledge, the first application of network analysis on the HAD and PSQI symptoms for PLWH.

First of all, the network model in the present study provided a new perspective on understanding the co-occurrence of mental illness for PLWH. We found that anxiety, depression, and sleep disturbance were entangled in the networks. Especially, anxiety and depression symptoms were strongly interconnected in all networks, aligning with the conclusion of previous studies that comorbidity between depressive and anxiety was common (34, 35). This finding implied that anxiety acted as a trigger for depression, and if symptoms of anxiety were present, the risk of the onset of symptoms of the depression might rise correspondingly, and vice versa (36). On the other hand, among all subgroups, connections between symptoms within each disorder were higher than connections between disorders. The reason might be that symptoms describing a disorder belonged to the same questionnaire, and often co-occurred and closely connected in the network. This was consistent with the finding of a previous study (35). We also found that nodes S11 ‘*medicine*’, A3 ‘*worried*’, and D3 ‘*cheerful*’ were bridge symptoms that could spread activation from one disorder to another. Such bridge symptoms are essential in the development and maintenance of comorbidities (37). As a result, psychiatrists may prevent and treat comorbidities from the perspective of bridge symptoms for PLWH.

In addition, the comparison of nine pairs of networks revealed some noteworthy similarities as well as marked differences, which might provide valuable inspirations and future guidance for improving HIV persons' mental health. For example, participants that felt supported by society/family or disclosed HIV status to family members shown a significantly less likelihood of sleep disturbance. And the two population's sleep symptoms were almost completely independent of symptoms of HAD in network models. This was an inspiring finding because a more sparsely connected network of symptoms indicated a better prognosis from a clinical standpoint (38). Thus building a harmonious and friendly social environment was the first and most important step to alleviate mental disorders of the HIV-infected persons.

In contrast, in a tightly connected network, activation of one symptom could quickly spread to other symptoms, leading to more symptoms over time. In this study, females and student infectors shown densely connected networks, possibly because the two populations were usually with vulnerable psychological states and huge mental burdens. Under such circumstances, living and coping with HIV became more challenging compared to other subpopulations. As a consequence, females and student infectors were easy to suffer quality of sleep, anxiety, and depression. More attention and closer watch should be paid to such populations.

Our study also found that some symptoms shown consistent high strength among all networks. These symptoms thus had a higher probability of triggering other symptoms and predicting a clinical outcome (30). For example, if someone developed a central symptom such as A7 *(I get sudden feelings of panic)*, he was more likely to develop other symptoms than those who developed a peripheral symptom such as S11 *(During the past month, how often have you taken medicine (prescribed or “over the counter”) to help you sleep?)* (32). An intervention targeting such symptoms would have the greatest potential to push the entire network into a healthier state and affect the status of an individual's mental state, whereas a low centrality symptom would be of limited therapeutic use (17). Therefore, in this study, symptoms with strong strength, namely A7, S2, and S4, were important symptoms and signs for predicting mental disorders as well as promising candidates for the intervention of PLWH.

On the other hand, some symptoms differed considerably in their node predictability across subgroups, aligning with previous studies that the psychometric properties of symptoms usually differed across subgroups (17, 39). Predictability can reflect the controllability of the network. That is, for a node with high predictability, we can control it via its neighboring nodes. On the contrary, when the predictability of a node is low, we can directly intervene in itself (33). In this study, S4 ‘*comfortless breath*’ showing lower node predictability for participants with support from society/family than those without support. Thus, an intervention on sleeping disordered breathing problems may be effective for the former subpopulation. These results inspired psychiatrists and clinicians that personalized invention to a particular subgroup was essential and might be more effective during treatment than adopting the same therapeutic schedule.

Moreover, we found that much symptom pairs varied in whether and how strongly they related to each other in different subgroups. For example, the edge between nodes S5 *(Cough or snore loudly)* and D7 *(I can enjoy a good book or radio or TV program)* ranged from strong for females to absent for other subgroups. Based on the network perspective, two symptoms with a strong connection could influence each other directly (30). Thus a symptom's deterioration might lead to a series of adverse progression, and vice versa. When this sort of strong/absent connection solely occurred in a specific subgroup such as S5-D7 abovementioned, psychiatrists could intervene on this subgroup specifically instead of applying the same treatment globally. However, it was still unclear which symptoms caused another and why symptoms pairs varied in connections in different networks. Future research may shed more light on the mechanisms of symptoms association's variance among different subgroups.

Furthermore, some of these results, to our knowledge, are exclusively observed in the network of the current study. For instance, a prior study (35) reported that ‘worried’ was the most central anxiety symptom to the network built for enrolled participants. However, in this study, ‘worried’ was not central across all networks built for subpopulations, and anxiety symptom ‘panic’ was the most central one based on PLWH. Briganti et al., (16) found that the networks built for females and males' depression symptoms were substantially similar regarding global strength, network structure, and edge values, whereas the networks estimated for the two subpopulations differed a lot in our experiments for PLWH. Such findings may be unique in the current study because patients infected with HIV usually suffered more difficult situations compared to the general population, such as facing discrimination, perceiving stigma, lacking support, and having sexual problems. Therefore, we used the network analysis in conjunction with subgroup analysis herein, aiming to provide more personalized advice for treating mental disorders of PLWH.

The current study also had several limitations. First, the present network models reflected the average results over many individuals belonging to the same subgroup, which could not provide person-level instructions for psychiatrists. Second, four out of 18 items in the PSQI questionnaire were excluded from the network analyses. This omission might substantially alter the relationships among nodes. Finally, we could not make casual inferences from results based on this cross-sectional dataset. The longitudinal data will be collected and used to infer whether a given symptom causes another symptom in the future study.

## Conclusions

We conducted a network analysis based on the self-report data about HAD and PSQI of the HIV-infected persons in this study. The network analysis based on different subgroups revealed some noteworthy similarities as well as marked differences, giving new insight into relations among symptoms of mental disorders and inspiring psychiatrists and clinicians that personalized invention to a particular subgroup was essential and might be more effective during treatment than adopting the same therapeutic schedule. This network analysis had the potential to provide future guidance for the mental health treatment for PLWH.

## Data Availability Statement

The datasets presented in this article are not readily available because patient information needs to be kept confidential. Requests to access the datasets should be directed to Xiaojie Huang, huangxiaojie78@ccmu.edu.cn.

## Ethics Statement

This study was approved by the Beijing You'an Hospital institutional review board. All participants provided written informed consent to complete a survey and extract their clinical data from medical records.

## Author Contributions

HL and XH: conceptualization, writing—reviewing, and editing. NW: data curation, methodology, and writing—original draft preparation. MW and XX: data curation. TZ and HW: investigation. All authors contributed to the article and approved the submitted version.

## Funding

This work was supported by the National Natural Science Foundation of China (Grant Numbers 81971707) and the National Science and Technology Major Project of China during the 13th Five-year Plan Period (2017ZX10201101).

## Conflict of Interest

The authors declare that the research was conducted in the absence of any commercial or financial relationships that could be construed as a potential conflict of interest.

## Publisher's Note

All claims expressed in this article are solely those of the authors and do not necessarily represent those of their affiliated organizations, or those of the publisher, the editors and the reviewers. Any product that may be evaluated in this article, or claim that may be made by its manufacturer, is not guaranteed or endorsed by the publisher.

## References

[1] HIV Infected Persons. (2019). Available online at: http://www.gov.cn/xinwen/2019-12/01/content_5457448.htm (accessed July 28, 2021).

[2] AIDS-Related Deaths. (2020). Availble online at: http://www.nhc.gov.cn/jkj/s3578/202103/f1a448b7df7d4760976fea6d55834966.shtml (accessed July 29, 2021).

[3] Lu Niu Dan Luo Ying Liu Silenzio VMB Xiao S. The mental health of people living with HIV in China, 1998–2014: a systematic review. Plos ONE. (2016) 11:e0153489. 10.1371/journal.pone.0153489 27082749 PMC4833336

[4] Li J Mo PKH Kahler CW Lau JTF Du M Dai Y. Prevalence and associated factors of depressive and anxiety symptoms among HIV-infected men who have sex with men in China. Aids Care. (2015) 28:465–70. 10.1080/09540121.2015.1118430 26689341

[5] Olagunju AT Adeyemi JD Ogbolu RE Campbell EA. A study on epidemiological profile of anxiety disorders among people living with HIV/AIDS in a sub-Saharan Africa HIV clinic. AIDS Behav. (2012) 16:2192–7. 10.1007/s10461-012-0250-x 22772942

[6] Orza L Bewley S Logie CH Crone TE Moroz S Strachan S. How does living with HIV impact on women's mental health? voices from a global survey. J Int Aids Soc. (2015) 18(suppl 5):20289. 10.7448/IAS.18.6.20289 26643460 PMC4672402

[7] Charlson FJ Baxter AJ Cheng HG Shidhaye R Whiteford HA. The burden of mental, neurological, and substance use disorders in China and India: a systematic analysis of community representative epidemiological studies. Lancet. (2016) 388:376–89. 10.1016/S0140-6736(16)30590-6 27209143

[8] Phillips MR Zhang J Shi Q Song Z Ding Z Pang S. Prevalence, treatment, and associated disability of mental disorders in four provinces in China during 2001-05: an epidemiological survey. Lancet. (2009) 373:2041–53. 10.1016/S0140-6736(09)60660-7 19524780

[9] Huang X Meyers K Liu X Li X Zhang T Xia E. The double burdens of mental health among aids patients with fully successful immune restoration: a cross-sectional study of anxiety and depression in China. Front Psychiatry. (2018) 9:384. 10.3389/fpsyt.2018.00384 30197608 PMC6117419

[10] Rihs TA Begley K Smith DE Sarangapany J Callaghan A Kelly M. Efavirenz and chronic neuropsychiatric symptoms: a cross-sectional case control study. HIV Med. (2006) 7:544–8. 10.1111/j.1468-1293.2006.00419.x 17105514

[11] Moskowitz JT Carrico AW Duncan LG Cohn MA Cheung EO Batchelder A. Randomized controlled trial of a positive affect intervention for people newly diagnosed with HIV. J Consult Clin Psychol. (2017) 85:409–23. 10.1037/ccp0000188 28333512 PMC5398944

[12] Chen W-T Lee S-Y Shiu C-S Simoni JM Pan C Bao M. Fatigue and sleep disturbance in HIV-positive women: a qualitative and biomedical approach. J Clin Nurs. (2013) 22:1262–9. 10.1111/jocn.12012 23279292 PMC3625469

[13] Lee KA Gay C Portillo CJ Coggins T Davis H Pullinger CR. Types of sleep problems in adults living with HIV/AIDS J Clin Sleep Med. (2012) 8:67–75. 10.5664/jcsm.1666 22334812 PMC3266344

[14] Borsboom D Cramer AOJ. Network analysis: an integrative approach to the structure of psychopathology. Annu Rev Clin Psychol. (2013) 9:91–121. 10.1146/annurev-clinpsy-050212-185608 23537483

[15] Armour C Fried EI Deserno MK Tsai J Pietrzak RH. A network analysis of DSM-5 posttraumatic stress disorder symptoms and correlates in U.S. military veterans. J Anxiety Disord. (2017) 45:49–59. 10.1016/j.janxdis.2016.11.008 27936411

[16] Briganti G Scutari M Linkowski P. Network structures of symptoms from the zung depression scale. Psychol Rep. (2020) 124:1897–911. 10.1177/0033294120942116 32686585

[17] Rhemtulla M Friedb EI Aggen SH Tuerlinckx F Kendler KS Borsboom D. Network analysis of substance abuse and dependence symptoms. Drug Alcohol Depend. (2016) 161:230–7. 10.1016/j.drugalcdep.2016.02.005 26898186 PMC4861635

[18] Deserno MK Borsboom D Begeer S Geurts HM. Multicausal systems ask for multicausal approaches: a network perspective on subjective well-being in individuals with autism spectrum disorder. Autism. (2016) 21:960–71. 10.1177/1362361316660309 27539846

[19] Kessler RC Chiu WT Demler O Merikangas KR Walters EE. Prevalence, severity, and comorbidity of 12-month DSM-IV disorders in the national comorbidity survey replication Arch Gen Psychiatry. (2005) 62:617–27. 10.1001/archpsyc.62.6.617 15939839 PMC2847357

[20] Nock MK Hwang I Sampson NA Kessler RC. Mental disorders, comorbidity and suicidal behavior: results from the national comorbidity survey replication. Mol Psychiatry. (2010) 15:868–76. 10.1038/mp.2009.29 19337207 PMC2889009

[21] Zigmond AS Snaith RP. The hospital anxiety and depression scale: manual. Acta Psychiatr Scand. (1983) 67: 361–70. 10.1111/j.1600-0447.1983.tb09716.x 6880820

[22] Buysse DJ Reynolds CF III Monk TH Berman SR Kupfer DJ. The pittsburgh sleep quality index: a new instrument for psychiatric practice and research. Psychiatry Res. (1989) 28:193–213. 10.1016/0165-1781(89)90047-4 2748771

[23] Epskamp S Borsboom D Fried EI. Estimating psychological networks and their accuracy: a tutorial paper. Behav Res Methods. (2018) 50:195–212. 10.3758/s13428-017-0862-1 28342071 PMC5809547

[24] Friedman J Hastie T Tibshirani R. Glasso: Graphical Lasso- estimation of Gaussian Graphical Models. (2014). Available online at: https://cran.r-project.org/web/packages/glasso/index.html

[25] Haslbeck JMB Waldorp LJ. mgm: Estimating time-varying mixed graphical models in high-dimensional data. J Stat Softw. (2020) 93:3125. 10.18637/jss.v093.i08

[26] Epskamp S Fried EI. A tutorial on regularized partial correlation networks. Psychol Methods. (2018) 24:617–34. 10.1037/met0000167 29595293

[27] Freeman LC. Centrality in social networks conceptual clarification. Soc Netw. (1978) 1:215–39. 10.1016/0378-8733(78)90021-7

[28] Schmitt M Bonacich P. Power and centrality: a family of measures. Am J Sociol. (1987) 92:1170–82. 10.1086/228631

[29] Borgatti SP. Centrality and network flow. Soc Netw. (2005) 27:55–71. 10.1016/j.socnet.2004.11.008

[30] Opsahl T Agneessens F Skvoretz J. Node centrality in weighted networks: Generalizing degree and shortest paths. Soc Netw. (2010) 32:245–51. 10.1016/j.socnet.2010.03.006

[31] Bronchain J Chabrol H. Exploring the relationship between schizotypal traits and dispositional mindfulness from a network perspective. J Nerv Ment Dis. (2020) 208:608–12. 10.1097/NMD.0000000000001169 32229789

[32] Fried EI van Borkulo CD Cramer AOJ Boschloo L Schoevers RA Borsboom D. Mental disorders as networks of problems: a review of recent insights. Soc Psychiatry Psychiatr Epidemiol. (2017) 52:1–10. 10.1007/s00127-016-1319-z 27921134 PMC5226976

[33] Haslbeck JMB Fried EI. How predictable are symptoms in psychopathological networks? a reanalysis of 18 published datasets. Psychol Med. (2017) 47:2767–76. 10.1017/S0033291717001258 28625186

[34] Cramer AOJ Waldorp van der Maas HLJ Borsboom D. Comorbidity: a network perspective. Behav Brain Sci. (2010) 33:137–50. 10.1017/S0140525X09991567 20584369

[35] Beard C Millner AJ Forgeard MJC Fried EI Hsu KJ Treadway MT. Network analysis of depression and anxiety symptom relationships in a psychiatric sample. Psychol Med. (2016) 46:3359–69. 10.1017/S0033291716002300 27623748 PMC5430082

[36] Ren L Wang Y Wu L Wei Z Cui L-B Wei X. Network structure of depression and anxiety symptoms in Chinese female nursing students. BMC Psychiatry. (2021) 21:279. 10.1186/s12888-021-03276-1 34059013 PMC8168020

[37] Jones PJ Ma R McNally RJ. Bridge Centrality: a network approach to understanding comorbidity. Multivariate Behav Res. (2021) 56:353–67. 10.1080/00273171.2019.1614898 31179765

[38] van Borkulo C Boschloo L Borsboom D Penninx BWJH Waldorp LJ Schoevers RA. Association of symptom network structure with the course of depression. JAMA Psychiatry. (2015) 72:1219–26. 10.1001/jamapsychiatry.2015.2079 26561400

[39] Gillespie NA Neale MC Prescott CA Aggen SH Kendler KS. Factor and item-response analysis DSM-IV criteria for abuse of and dependence on cannabis, cocaine, hallucinogens, sedatives, stimulants and opioids. Addiction. (2010) 102:920–30. 10.1111/j.1360-0443.2007.01804.x 17523987

