# Supplementary material for: Exploring the Relationship Between Anxiety, Depression, and Sleep Disturbance Among HIV Patients in China From a Network Perspective
Source: Front Psychiatry. 2021 Oct 22;12:764246. doi: 10.3389/fpsyt.2021.764246 (PMC8569919; doi:10.3389/fpsyt.2021.764246)
Supplement: Supplementary file 1 [file Data_Sheet_1.docx]

# Supplementary materials

Table S1. Node predictability of items on the Hospital Anxiety and Depression (HAD) scale questionnaire in all subgroup networks.

| Nodes  Networks | HAD on anxiety | | | | | | | HAD on depression | | | | | | |
| --- | --- | --- | --- | --- | --- | --- | --- | --- | --- | --- | --- | --- | --- | --- |
|  | A1 | A2 | A3 | A4 | A5 | A6 | A7 | D1 | D2 | D3 | D4 | D5 | D6 | D7 |
| Age | | | | | | | | | | | | | | |
| Age, 18~35, years | 38% | 45% | 37% | 34% | 29% | 26% | 47% | 22% | 31% | 41% | 27% | 23% | 29% | 22% |
| Age, >35, years | 44% | 47% | 41% | 32% | 30% | 34% | 47% | 23% | 30% | 39% | 31% | 26% | 28% | 25% |
| Gender | | | | | | | | | | | | | | |
| Male | 40% | 45% | 39% | 31% | 28% | 31% | 47% | 23% | 31% | 40% | 28% | 25% | 27% | 23% |
| Female | 44% | 47% | 42% | 38% | 34% | 26% | 45% | 25% | 30% | 39% | 29% | 23% | 30% | 23% |
| Education | | | | | | | | | | | | | | |
| <High school | 44% | 48% | 35% | 33% | 28% | 30% | 46% | 23% | 28% | 37% | 31% | 22% | 25% | 20% |
| High school | 40% | 46% | 42% | 28% | 31% | 30% | 47% | 23% | 33% | 40% | 26% | 25% | 29% | 23% |
| >High school | 40% | 44% | 43% | 37% | 30% | 30% | 48% | 24% | 32% | 44% | 30% | 28% | 33% | 27% |
| Transmission route | | | | | | | | | | | | | | |
| Heterosexual | 44% | 47% | 38% | 31% | 29% | 31% | 46% | 24% | 30% | 39% | 29% | 23% | 27% | 21% |
| Homosexual | 37% | 46% | 39% | 34% | 27% | 29% | 49% | 21% | 34% | 40% | 29% | 24% | 28% | 26% |
| Martial status | | | | | | | | | | | | | | |
| Married | 42% | 46% | 41% | 29% | 32% | 29% | 45% | 23% | 31% | 38% | 25% | 23% | 27% | 24% |
| Single | 39% | 45% | 39% | 33% | 29% | 28% | 48% | 23% | 30% | 41% | 30% | 28% | 31% | 22% |
| Employment | | | | | | | | | | | | | | |
| Student | 48% | 50% | 45% | 37% | 32% | 27% | 47% | 31% | 32% | 43% | 33% | 25% | 30% | 20% |
| Blue collars | 41% | 49% | 37% | 32% | 28% | 34% | 49% | 19% | 29% | 37% | 26% | 25% | 26% | 24% |
| White collars | 37% | 42% | 39% | 35% | 29% | 28% | 43% | 21% | 34% | 39% | 30% | 27% | 29% | 26% |
| Disclosed to friends | | | | | | | | | | | | | | |
| Yes | 42% | 46% | 42% | 32% | 30% | 31% | 46% | 24% | 31% | 40% | 29% | 26% | 27% | 23% |
| No | 37% | 45% | 31% | 34% | 26% | 24% | 47% | 21% | 32% | 38% | 27% | 22% | 32% | 24% |
| Disclosed to family | | | | | | | | | | | | | | |
| Yes | 41% | 44% | 41% | 35% | 30% | 32% | 45% | 25% | 36% | 45% | 33% | 28% | 26% | 25% |
| No | 40% | 46% | 38% | 31% | 28% | 29% | 47% | 21% | 28% | 37% | 26% | 22% | 30% | 23% |
| Support from society/family | | | | | | | | | | | | | | |
| With | 38% | 45% | 37% | 32% | 28% | 28% | 45% | 22% | 31% | 38% | 26% | 23% | 29% | 23% |
| Without | 46% | 45% | 41% | 31% | 28% | 33% | 47% | 24% | 29% | 40% | 33% | 25% | 25% | 21% |
| Mean±standard deviation | 41% ± 2.9% | 46% ± 1.9% | 39% ± 3.1% | 33% ± 2.7% | 29% ± 2.0% | 29% ± 2.3% | 47% ± 1.5% | 23% ± 2.5% | 31% ± 2.2% | 40% ± 2.3% | 29% ± 2.3% | 25% ± 2.1% | 28% ± 2.3% | 23% ± 1.9% |

Table S2. Node predictability of items on the Pittsburgh Sleep Quality Index questionnaire in all subgroup networks.

Nodes

| Networks  Networks | S1 | S2 | S3 | S4 | S5 | S6 | S7 | S8 | S9 | S10 | S11 | S12 | S13 | S14 |
| --- | --- | --- | --- | --- | --- | --- | --- | --- | --- | --- | --- | --- | --- | --- |
| Age | | | | | | | | | | | | | | |
| Age, 18~35, years | 37% | 41% | 18% | 19% | 11% | 20% | 19% | 23% | 19% | 22% | 5% | 36% | 30% | 35% |
| Age, >35, years | 40% | 42% | 19% | 25% | 11% | 24% | 24% | 19% | 21% | 22% | 7% | 42% | 36% | 41% |
| Gender | | | | | | | | | | | | | | |
| Male | 37% | 41% | 19% | 21% | 11% | 19% | 19% | 19% | 19% | 22% | 6% | 39% | 31% | 37% |
| Female | 44% | 42% | 19% | 21% | 14% | 26% | 22% | 25% | 24% | 23% | 7% | 40% | 42% | 41% |
| Education | | | | | | | | | | | | | | |
| <High school | 39% | 41% | 22% | 23% | 12% | 25% | 25% | 20% | 22% | 22% | 7% | 42% | 36% | 40% |
| High school | 38% | 42% | 19% | 22% | 14% | 24% | 23% | 25% | 23% | 24% | 6% | 37% | 30% | 33% |
| >High school | 39% | 41% | 18% | 18% | 9% | 16% | 17% | 20% | 17% | 24% | 4% | 38% | 32% | 40% |
| Transmission route | | | | | | | | | | | | | | |
| Heterosexual | 38% | 38% | 18% | 17% | 10% | 20% | 19% | 17% | 18% | 20% | 4% | 43% | 36% | 38% |
| Homosexual | 37% | 44% | 18% | 17% | 9% | 17% | 16% | 20% | 17% | 22% | 6% | 35% | 28% | 35% |
| Martial status | | | | | | | | | | | | | | |
| Married | 38% | 40% | 18% | 20% | 11% | 22% | 23% | 18% | 21% | 18% | 8% | 39% | 31% | 36% |
| Single | 40% | 44% | 19% | 21% | 10% | 19% | 18% | 22% | 23% | 24% | 5% | 37% | 32% | 38% |
| Employment | | | | | | | | | | | | | | |
| Student | 45% | 48% | 22% | 29% | 11% | 21% | 26% | 22% | 25% | 28% | 7% | 44% | 40% | 40% |
| Blue collars | 35% | 38% | 18% | 18% | 10% | 23% | 21% | 19% | 18% | 20% | 5% | 40% | 33% | 36% |
| White collars | 41% | 40% | 18% | 21% | 11% | 22% | 19% | 23% | 21% | 26% | 8% | 36% | 28% | 40% |
| Disclosed to friends | | | | | | | | | | | | | | |
| Yes | 40% | 42% | 20% | 20% | 10% | 22% | 22% | 20% | 20% | 22% | 6% | 39% | 33% | 38% |
| No | 33% | 41% | 19% | 23% | 12% | 19% | 16% | 22% | 21% | 21% | 5% | 39% | 33% | 35% |
| Disclosed to family | | | | | | | | | | | | | | |
| Yes | 40% | 42% | 18% | 23% | 10% | 21% | 19% | 22% | 23% | 21% | 7% | 37% | 36% | 36% |
| No | 37% | 41% | 19% | 20% | 12% | 21% | 21% | 20% | 19% | 23% | 5% | 40% | 32% | 38% |
| Support from society/family | | | | | | | | | | | | | | |
| With | 37% | 41% | 19% | 16% | 9% | 19% | 18% | 19% | 19% | 21% | 5% | 37% | 32% | 36% |
| Without | 42% | 42% | 20% | 31% | 14% | 23% | 25% | 23% | 22% | 23% | 8% | 41% | 36% | 38% |
| Mean±standard deviation | 39% ± 2.9% | 42% ± 2.3% | 19% ± 1.2% | 21% ± 2.9% | 11% ± 1.5% | 21% ± 2.7% | 20% ± 3.0% | 21% ± 2.2% | 21% ± 2.4% | 22% ± 2.2% | 6% ± 1.2% | 39% ± 2.3% | 33% ± 3.8% | 38% ± 2.3% |


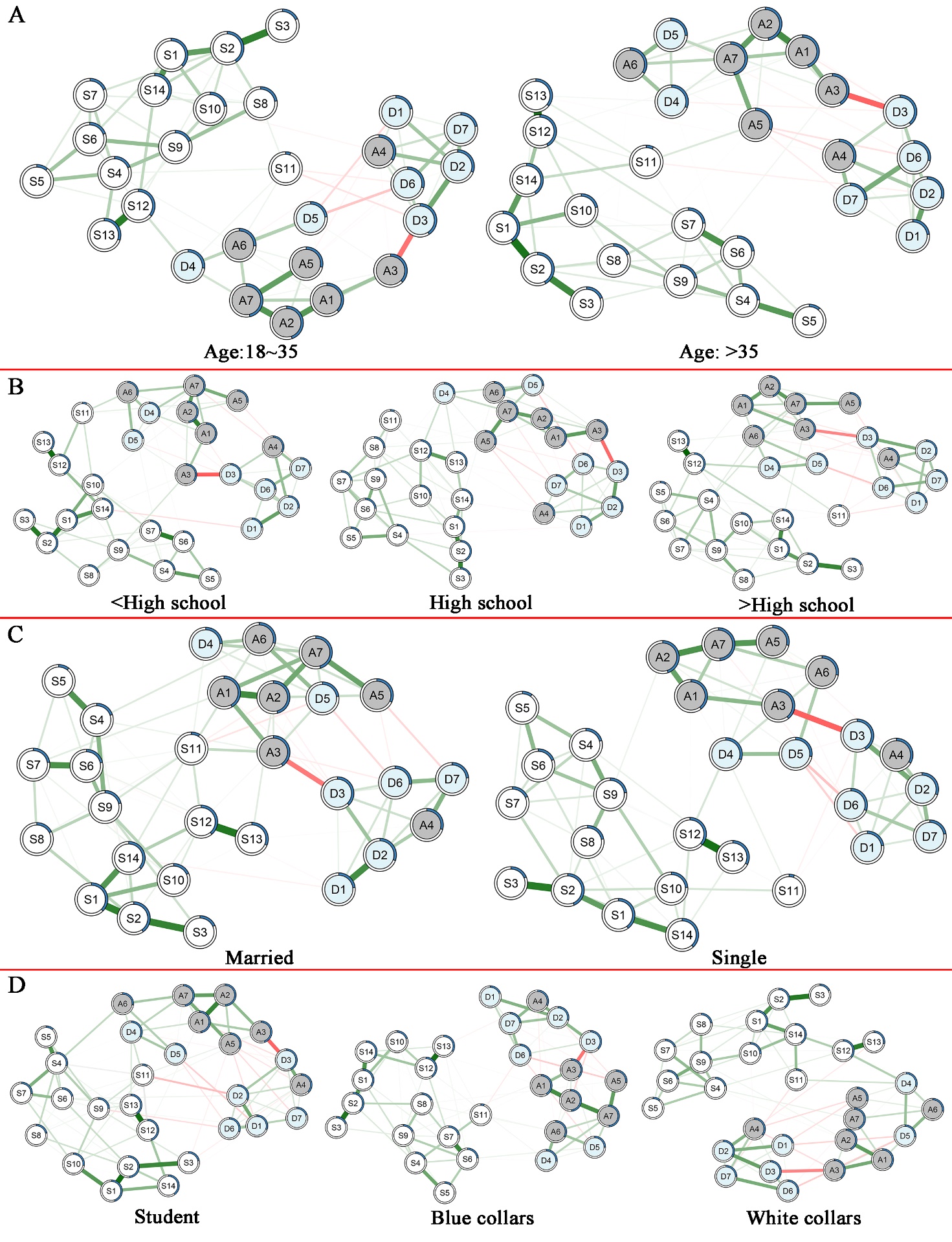


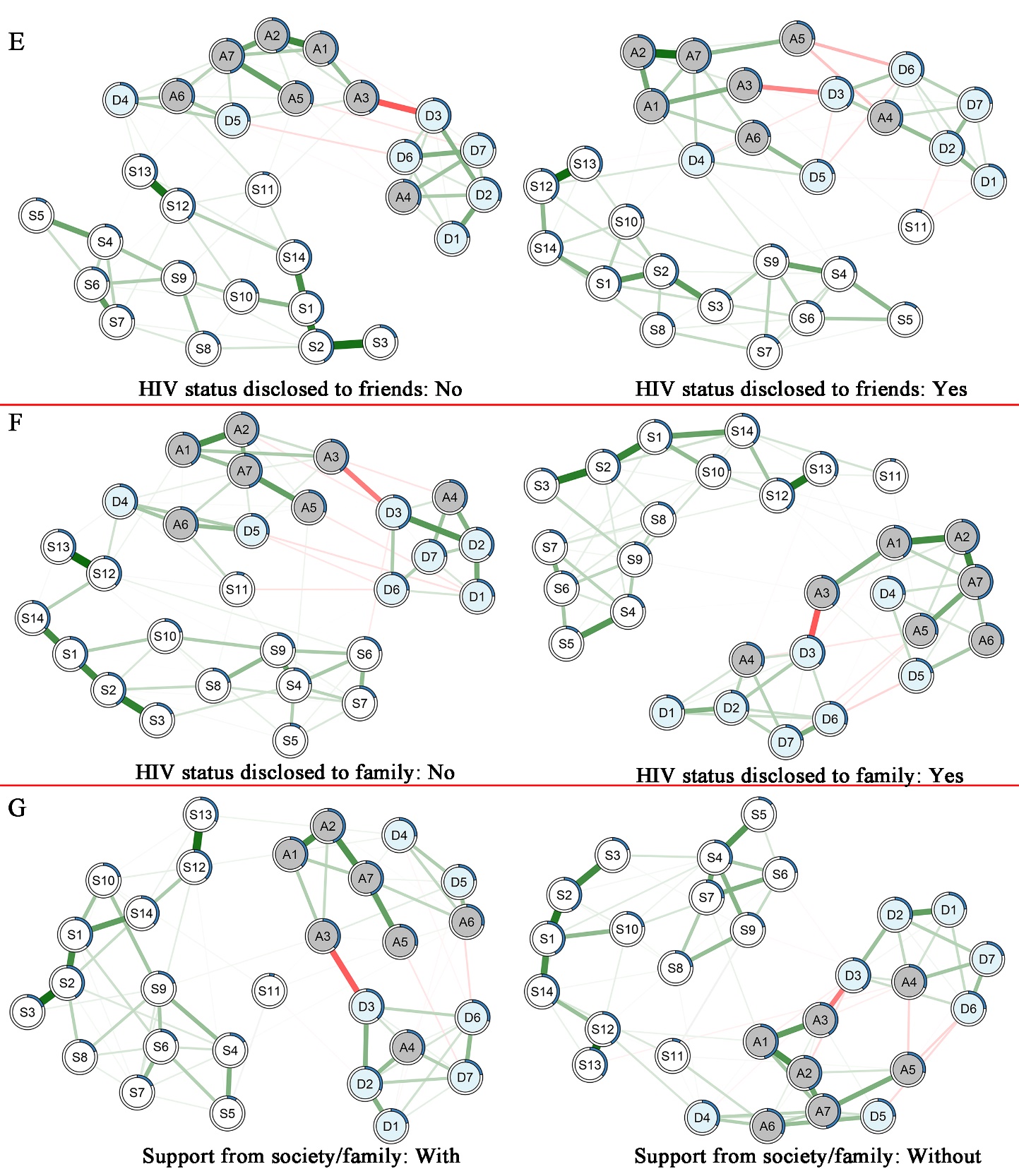


Fig. S1 Networks featuring 28 symptoms on the Hospital Anxiety and Depression scale questionnaire and the Pittsburgh Sleep Quality Index questionnaire for A) age; B) education; C) marital status; D) employment; E) HIV status disclosed to friends; F) HIV status disclosed to family; G) Support from society /family.
